# Quadruped Gait and Regulation of Apoptotic Factors in Tibiofemoral Joints following Intra-Articular rhPRG4 Injection in *Prg4* Null Mice

**DOI:** 10.3390/ijms23084245

**Published:** 2022-04-12

**Authors:** Daniel S. Yang, Edward E. Dickerson, Ling X. Zhang, Holly Richendrfer, Padmini N. Karamchedu, Gary J. Badger, Tannin A. Schmidt, Alger M. Fredericks, Khaled A. Elsaid, Gregory D. Jay

**Affiliations:** 1 School of Engineering, Brown University, Providence, RI 02912, USA; daniel_yang1@brown.edu (D.S.Y.); gregory_jay_md@brown.edu (G.D.J.); 2 Department of Emergency Medicine, Alpert School of Medicine, Brown University, Providence, RI 02903, USA; lzhang@lifespan.org (L.X.Z.); holly.richendrfer@gmail.com (H.R.); 3 North Carolina Agricultural Technical State University, Greensboro, NC 27411, USA; eddiedickerson5@gmail.com; 4 Department of Orthopedics, Alpert School of Medicine, Brown University, Providence, RI 02903, USA; minikaramchedu@gmail.com; 5 Department of Medical Biostatistics, Larner College of Medicine, University of Vermont, Burlington, VT 05405, USA; gbadger@uvm.edu; 6 Department of Biomedical Engineering, School of Dental Medicine, University of Connecticut Health, Farmington, CT 06030, USA; tschmidt@uchc.edu; 7 Department of Surgery, Alpert School of Medicine, Brown University, Providence, RI 02903, USA; alger_fredericks@brown.edu; 8 School of Pharmacy, Chapman University, Irvine, CA 92618, USA

**Keywords:** PRG4, lubricin, CACP, arthrosis, arthritis, rhPRG4, *Prg4* ^−/−^, collagen hybridizing peptide

## Abstract

Camptodactyly-arthropathy-coxa vara-pericarditis (CACP) syndrome leads to diarthrodial joint arthropathy and is caused by the absence of lubricin (proteoglycan 4—PRG4), a surface-active mucinous glycoprotein responsible for lubricating articular cartilage. In this study, mice lacking the orthologous gene *Prg4* served as a model that recapitulates the destructive arthrosis that involves biofouling of cartilage by serum proteins in lieu of Prg4. This study hypothesized that Prg4-deficient mice would demonstrate a quadruped gait change and decreased markers of mitochondrial dyscrasia, following intra-articular injection of both hindlimbs with recombinant human PRG4 (rhPRG4). *Prg4*^−/−^ (N = 44) mice of both sexes were injected with rhPRG4 and gait alterations were studied at post-injection day 3 and 6, before joints were harvested for immunohistochemistry for caspase-3 activation. Increased stance and propulsion was shown at 3 days post-injection in male mice. There were significantly fewer caspase-3-positive chondrocytes in tibiofemoral cartilage from rhPRG4-injected mice. The mitochondrial gene *Mt-tn*, and myosin heavy (*Myh7*) and light chains (*Myl2* and *Myl3*), known to play a cytoskeletal stabilizing role, were significantly upregulated in both sexes (RNA-Seq) following IA rhPRG4. Chondrocyte mitochondrial dyscrasias attributable to the arthrosis in CACP may be mitigated by IA rhPRG4. In a supporting in vitro crystal microbalance experiment, molecular fouling by albumin did not block the surface activity of rhPRG4.

## 1. Introduction

Superficial zone chondrocytes and synoviocytes express lubricin (proteoglycan 4—PRG4), a highly surface-active mucinous glycoprotein responsible for lubricating articular cartilage. In addition to reducing friction, PRG4 functions to reduce cell adhesion, inhibit synovial cell overgrowth and hyperplasia, and prevent cartilage–cartilage integration [1,2]. Camptodactyly-arthropathy-coxa vara-pericarditis (CACP) syndrome is caused by a deficiency in PRG4 inherited as a recessive trait from both parents [3]. These patients have variable joint involvement, and report joint pain early in the first decade, which increases after 10 years of age [4]. Mice lacking the orthologous gene *Prg4* may serve as an arthrosis model [5] to understand the joint failure attributable to lack of PRG4 in humans, since many clinical features are recapitulated [6].

In *Prg4*^−/−^ [6] and *Prg4*^GT/GT^ [3] mice, Prg4 deficiency results in increased joint friction [7], synovial hyperplasia [6], and superficial and intermediate zone apoptosis [8,9]. In humans, CACP leads to precocious cartilage failure [10,11]. CACP may also be considered an arthropathy that is partially inflammation mediated, as elucidated in animal models of Prg4 deficiency and as patients with CACP have used non-steroidal anti-inflammatory medications for pain relief [12,13,14]. Mutant *Prg4* mouse models show cartilage surface damage as early as 2 weeks of age [7] and increased whole joint friction by 2 months, indicating alteration in the whole joint mechanics early in life [15]. Prg4-deficient joints may experience elevated friction from articular biofouling with albumin and other synovial fluid non-lubricating proteins [6]. Reactive oxygen species are generated from chondrocytes subjected to friction-induced mechanical shear [8,16].

Studies in the Yucatan minipig have demonstrated the efficacy of intra-articular (IA) injection of recombinant human lubricin (rhPRG4) to reduce collagen type II degradation, cartilage damage, and interleukin 1 beta (IL-1β) levels locally and systemically following a medial meniscus injury [5]. A within-group analysis of *Prg4*^−/−^ mice receiving rhPRG4 via IA injection found lower whole joint friction in the injected knee compared to the contralateral joint [8]. PRG4-deficient synovial fluid from patients with chronic osteoarthritis (OA) had significantly diminished cartilage boundary-lubricating ability, which was restored when supplemented with PRG4 purified from bovine explants, in a cartilage-on-cartilage friction test [17]. The IA injection of human synoviocyte PRG4 in ACL-transected rat joints restored symmetrical gait compared to transected rats that only received phosphate-buffered saline (PBS) IA injection [18]. A characteristic limitation of these studies is that typically, only one joint is affected or injected.

Changes in gait have previously been reported in a case report of CACP involving facet joint arthropathy and bony ankylosis [19]. The characterization of gait modifications has been explored in OA, where pain and compensation in gait protect injured limbs from loading, preventing loss of articular cartilage and formation of osteophytes by altering internal joint mechanics [20]. Given the important role of PRG4 in boundary lubrication and joint biomechanics, we sought to characterize the gait disturbances attributable to a lack of Prg4 in an orthologous mouse model. This study hypothesized that *Prg4*^−/−^ mice would demonstrate a reversible quadruped gait change accompanied by reversal of mitochondrial stress in apoptotic chondrocytes, as assessed by RNA-Seq and immunohistochemistry following rhPRG4 injection of both hindlimbs. This study served as an initial effort to determine if IA rhPRG4 holds any therapeutic promise in treating CACP in an appropriate animal model that shares several clinical features of CACP syndrome [6].

## 2. Results

### 2.1. Gait and Posture Indices of Prg4^−/−^ and Prg4^+/+^ Mice

Significant differences in gait were found between *Prg4*^−/−^ and *Prg4*^+/+^ male mice in five gait parameters (Table 1). Male *Prg4*^−/−^ mice had a decreased percent of stride in braking (*p* < 0.001), increased percent of stride in propulsion (*p* < 0.001), increased swing time (*p* = 0.048), decreased brake time (*p* < 0.001), and increased propel time (*p* < 0.001), compared to male *Prg4*^+/+^ mice. In female *Prg4*^−/−^ and *Prg4*^+/+^ mice, significant differences were found in five parameters (Table 1). Female *Prg4*^−/−^ mice also showed an increased percent of stride in propulsion (*p* = 0.007), increased swing time (*p* < 0.001), and decreased brake time (*p* < 0.001), compared to female *Prg4*^+/+^ mice. Female *Prg4*^−/−^ mice showed a decrease in stance time (*p* < 0.001) and stride time (*p* < 0.001) compared to *Prg4*^+/+^ female littermates, which is different from the male *Prg4*^−/−^ mice, which showed increases in these parameters.

Other gait parameters that did not show significant changes in comparing *Prg4*^−/−^ and *Prg4*^+/+^ mice in either sex were percent of stride in swing, and stance divided by swing. However, there were significant groupwise comparisons in these parameters across sexes (Table 1) since percent of stride in swing was larger for *Prg4*^−/−^ male mice and smaller for *Prg4*^−/−^ female mice. Similarly, stance divided by swing was larger for *Prg4*^−/−^ female mice and smaller for *Prg4*^−/−^ male mice.

#### Changes in Gait and Posture Indices following rhPRG4 Injection in *Prg4*^−/−^ Mice

In the analysis of the *Prg4*^−/−^ mice gait data, rhPRG4 injection (N = 12) led to increased time in the stance phase from baseline to day 3 post-injection compared to mice (N = 12) that received the PBS injection (*p* = 0.005). rhPRG4 injection also led to an increased propel time at day 3 compared to baseline, versus PBS injection (*p* = 0.020) (Table 2a). Differences between day 6 post-injection and baseline gait showed no statistical difference between rhPRG4- and PBS-injected animals. Overall, these results indicate that the male mice were able to spend more time in stance and in propulsion during their gait. Changes in gait parameters following IA rhPRG4 were not observed in female mice by day 3 or 6 following injection (Table 2b).

### 2.2. Caspase-3 Activation in Superficial Zone Chondrocytes

**Figure 1:**
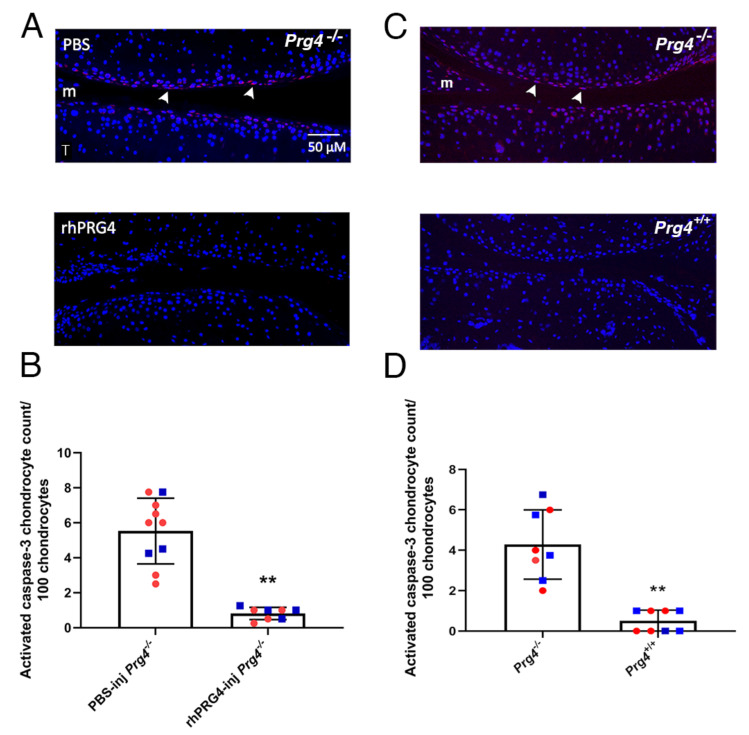
Suppression of caspase-3 activation by rhPRG4 in *Prg4*^−/−^ mouse joints. (**A**) Immunostaining for active caspase-3 in representative knee joint coronal sections of 8-week-old *Prg4*^−/−^ mice that received IA rhPRG4 or PBS 10 days prior. Note the immunopositivity in superficial zone chondrocytes (arrow heads) that received PBS contrasted with littermates that received rhPRG4 and showed a smaller signal. The application of rhPRG4 in *Prg4*^−/−^ joints serves to reduce mechanical shear stress in both diseased and healthy cartilage. F = femoral condyle and m = meniscus. (**B**) Counts of chondrocytes with caspase-3 activation per 100 chondrocytes from both the femoral and tibial cartilage computed across 10 mice (3**♂**■ and 7**♀**■) treated with PBS and 8 mice (4**♂**■ and 4**♀**■) treated with rhPRG4 10 days after IA injection. (**C**) Immunostaining for active caspase-3 in chondrocytes (arrow heads) in representative knee joint coronal sections of 8-week-old non-injected control *Prg4*^−/−^ and *Prg4*^+/+^ mice. (**D**) Corresponding counts of chondrocytes with caspase-3 activation in 8-week-old non-injected control *Prg4*^−/−^ (4**♂**■ and 4**♀**■) and *Prg4*^+/+^ mice (4**♂**■ and 4**♀**■). Mean ± SD displayed. ** *p* < 0.01.

Immunostaining of joints from male and female *Prg4*^−/−^ mice for active caspase-3 showed significantly lower immunopositivity in superficial zone chondrocytes from the knee joint coronal sections of mice that received rhPRG4 IA injection (N = 4 males and N = 4 females) compared to PBS injection (N = 3 males and N = 7 females) at 10 days (*p* < 0.01). For comparison, activated caspase-3 was readily detected in non-injected control *Prg4*^−/−^ mice in both sexes. In contrast, *Prg4*^+/+^ mice of both sexes show little or no signal (Figure 1).

### 2.3. RNA-Seq Analysis of Recovered Articular Cartilage in Male and Female Prg4^−/−^ Mice following rhPRG4 Injection

**Figure 2:**
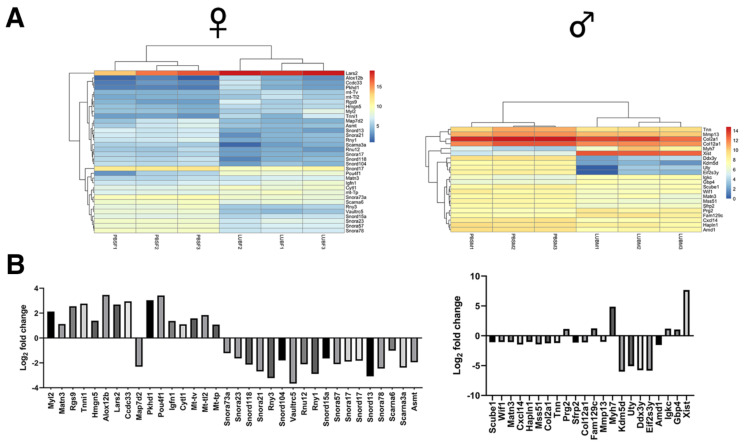
RNA-Seq analysis of chondrocytes recovered from individual male and female *Prg4*^−/−^ mice treated with either rhPRG4 or PBS. (**A**) Bi-clustering heat map of differentially expressed genes assorted by their adjusted *p*-values for both sexes and (**B**) Log2 fold change in genes in PBS vs. rhPRG4 from RNA-seq data for both sexes. Heatmap colors represent the log2 expression values of each group for each gene. Groups were clustered based upon their similar expression values and presented as dendrograms. In total, 34 and 22 known significantly differentially expressed genes were identified in female and male *Prg4*^−/−^ mice, respectively.

The statistics of the mapping of reads to the reference genome are illustrated in Supplementary Table S1. In comparing male rhPRG4- and PBS-injected mice, 22 significantly differentially expressed genes were identified, as illustrated in Figure 2 and identified in Supplementary Table S2. Genes that were upregulated in male rhPRG4-injected mice compared to PBS mice were *Prg2*, *Fam129c*, *Myh7*, *Igkc*, *Gbp4*, and *Xist.* Downregulated genes were *Scube1*, *Wif1*, *Matn3*, *Cxcl14*, *Hapln1*, *Mss51*, *Col2a1*, *Tnn*, *Sfrp2*, *Col12a1*, *Mmp13*, *Kdm5d*, *Uty*, *Ddx3y*, *Eif2s3y*, and *Amd1.* Selected confirmatory qRT-PCR for *Myh7* and *Xist* showed a relative fold expression change of 24.8 and 51.8, respectively, compared to cartilage from control tibio-femoral joints that received IA PBS.

In comparing female rhPRG4- and PBS-injected mice, there were 34 significantly differentially expressed genes, as illustrated in Figure 2 and identified in Supplementary Table S2. Upregulated genes in female rhPRG4-injected mice compared to PBS mice were *Myl2*, *Matn3*, *Rgs9*, *Tnni1*, *Hmgn5*, *Alox12b*, *Lars2*, *Ccdc33*, *Pkhd1*, *Pou4f1*, *Igfn1*, *Cytl1*, *Mt-tv*, *Mt-tl2*, and *Mt-tp*. Downregulated genes were *Mapt7d2*, *Snora73a*, *Snora23*, *Snord118*, *Snora21*, *Rny3*, *Snord104*, *Vaultrc5*, *Rnu12*, *Rny1*, *Snord15a*, *Snora57*, *Snora17*, *Snord17*, *Snord13*, *Snora78*, *Scarna6*, *Scarna3a*, and *Asmt*. Selected confirmatory qRT-PCR for *Lars2*, *Myl2*, and *Tnni1* showed a relative fold expression change of 5.4, 3350.2, and 950.6, respectively, compared to cartilage from control tibio-femoral joints that received IA PBS.

**Figure 3:**
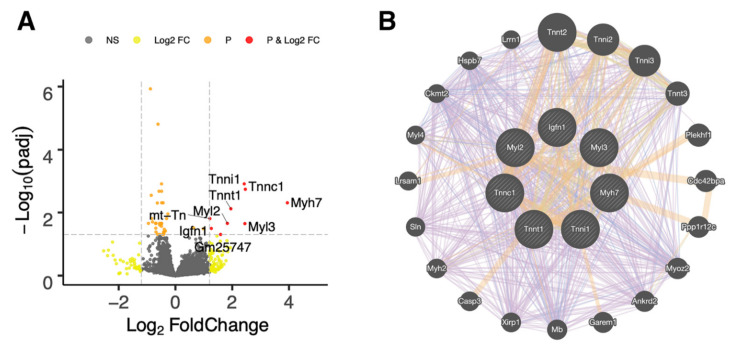
Analysis of merged RNA-Seq data (both sexes PBS vs. rhPRG4) of chondrocytes from male and female *Prg4*^−/−^ mice of differentially expressed genes, assorted by their adjusted *p* (padj) values independent of sex, treated with rhPRG4 compared to PBS. (**A**) Volcano plot of differentially expressed genes, where padj < 0.05 genes are labeled in red. (**B**) GeneMANIA Cytoscape interaction network of genes associated with the significantly upregulated core node genes (cross-hatched circles). Line color depicts gene co-expression (purple), predicted association (orange), and co-localization (blue). Supplementary Tables S2 and S3 illustrate the associated gene identities, which include cytoskeletal and mitochondrial genes, associated with mitochondrial metabolism or associated with mitochondrial DNA. Troponin I1 (Tnni1) has been previously associated with CACP syndrome in humans. Note that *mt-Tn* and Gm25747 (predicted gene) were not selectable in the GeneMANIA database and thus not incorporated as core node genes.

In pooled-sex rhPRG4- and PBS-injected mice, there were several significantly differentially expressed upregulated genes, as illustrated by the Volcano plot (Figure 3A) and identified in Supplementary Table S2. These included *Myh7*, *Mt-tn*, *Igfn1*, *Myl2*, *Myl3*, *Tnnc1*, *Tnni1*, and *Tnnt1*.

### 2.4. qRT-PCR of Nerve Growth Factor and Apoptotic Genes from Recovered Synovium

**Figure 4:**
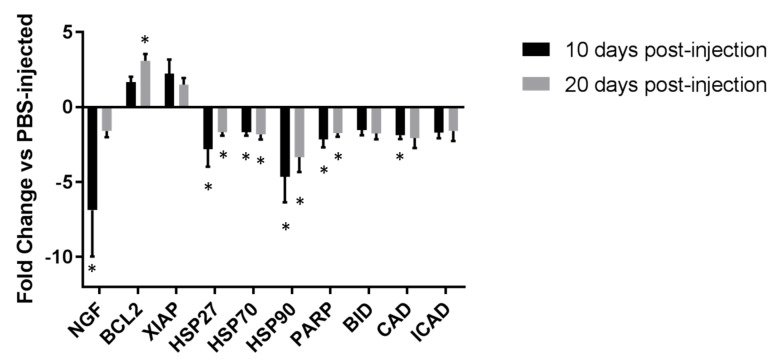
Quantitative real-time PCR of apoptosis mediators in synoviocytes from rhPRG4-injected *Prg4*^−/−^ mouse knees. The ∆∆Ct calculation represented as a log2 fold change relative to *Prg4*^−/−^ littermates injected with PBS. Synovium samples were pooled from rhPRG4-injected N = 3 and PBS-injected N = 3 mice at 10 and 20 days following IA injection. Mean ± SEM displayed. * *p* < 0.05.

The synovium of *Prg4*^−/−^ mice injected IA with rhPRG4 as opposed to PBS had decreased expression of nerve growth factor (*Ngf*) at day 10 (*p* = 0.006) but not day 20 (*p* = 0.104) (Figure 4). The expression of B cell lymphoma 2 (*Bcl2*) was increased by rhPRG4 injection by day 20 post-injection (*p* = 0.001), with differences at day 10 post-injection that trended towards significance (*p* = 0.065). Interestingly, the expression of heat shock proteins 27, 70, and 80 (*Hsp27*, *Hsp70*, *Hsp90*) were significantly decreased at both day 10 (*Hsp27 p* = 0.042, *Hsp70 p* = 0.017, *Hsp90 p* = 0.008) and day 20 (*Hsp27 p* = 0.019, *Hsp70 p* = 0.025, *Hsp90 p* = 0.009) in synovium from rhPRG4-injected animals compared to PBS-injected animals. Poly(ADP)-ribose polymerase-1 (*Parp*) and caspase-activated DNase (*Cad*) were both significantly decreased in the synovium of rhPRG4- vs. PBS-injected animals at days 10 (*p* = 0.028) and 20 (*p* = 0.011) for PARP and at day 10 for CAD (*p* = 0.007). BH3 interacting-domain death agonist (*Bid*) decreased at day 20 and approached significance (*p* = 0.056).

### 2.5. Accumulation of Collagen-Binding Protein in the Superficial Zone

Probing the IA-injected *Prg4*^−/−^ tibio-femoral joints with a collagen-binding peptide [21] sourced from 3Helix (Salt Lake, UT, USA) revealed more binding and thus degradation of collagen throughout the joint. This was especially pronounced qualitatively in the superficial zone (Supplementary Figure S1). Segmenting the fluorescence signal and optimizing the quantitation [22] in the superficial zone to a depth of 10 μm from the surface, and across the femoral and tibial condylar surfaces revealed that the rhPRG4-treated *Prg4*^−/−^ joints from combined male and female mice showed significantly less degradation or overturn of collagen.

### 2.6. Quartz Microbalance Measurements for Adsorption and Anti-Adhesion

**Figure 5:**
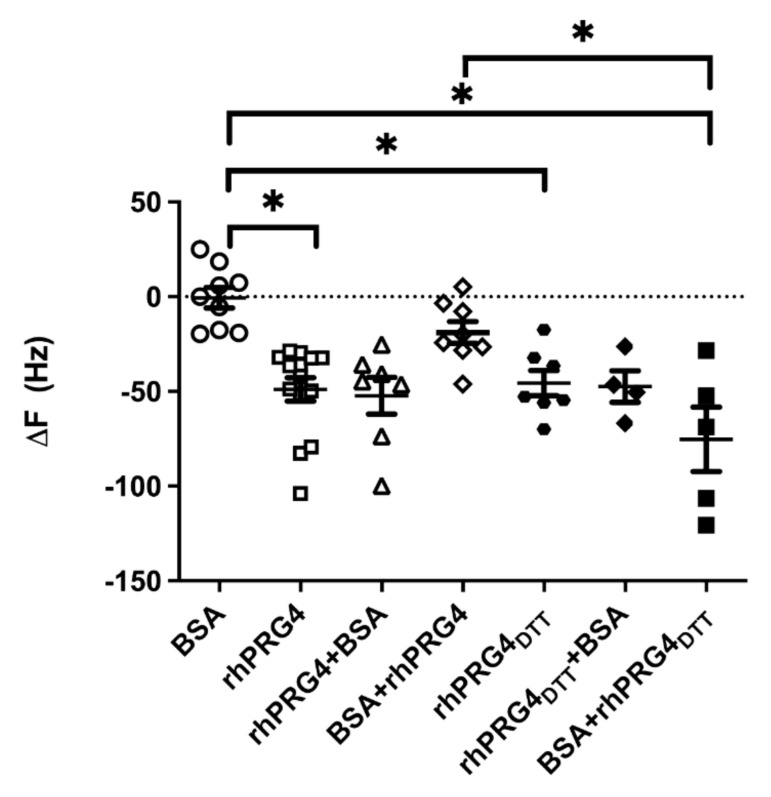
Anti-adhesive activity of rhPRG4 and DTT-treated rhPRG4 (rhPRG4_DTT_) in the quartz crystal microbalance. Mean ΔF values, a measure of the amount of adsorption on the crystal surface, of BSA, rhPRG4, and rhPRG4_DTT_ are shown along with ΔF measurements on surfaces coated with either rhPRG4 or rhPRG4_DTT_ followed by BSA or vice versa. Mean ΔF was unaffected by BSA compared to rhPRG4 (*p* < 0.05) and rhPRG4_DTT_ (*p* < 0.05). Mean ΔF of rhPRG4 was not significantly different from rhPRG4_DTT_ (*p* > 0.05). Mean ΔF of BSA on an rhPRG4 coated surface was not significantly different from rhPRG4 adsorption alone (*p* > 0.05). Mean ΔF of rhPRG4_DTT_ on a BSA-coated surface (BSA + rhPRG4_DTT_) was significantly lower compared to BSA (*p* < 0.05). Mean ± SD displayed. * *p* < 0.05.

The mean ΔF of bovine serum albumin (BSA) (−0.53 ± 16.0 Hz) was significantly lower than ΔF for rhPRG4 (−49.0 ± 23.0 Hz) and rhPRG4_DTT_ (−46.0 ± 18.0 Hz) (*p* < 0.05), showing that both preparations of rhPRG4 were more surface active than BSA (Figure 5). No significant differences were observed between rhPRG4 alone and rhPRG4 followed by BSA or for rhPRG4_DTT_ followed by BSA. The mean ΔF of BSA followed by rhPRG4_DTT_ (−75.0 ± 38.0 Hz) (*p* < 0.05) was significantly different from BSA. The ΔF of BSA followed by rhPRG4 (−19.0 ± 16.0 Hz) was not as large and significantly different (*p* < 0.05) (Figure 5). The dithiothreitol (DTT) treatment of rhPRG4 served to monomerize PRG4.

## 3. Discussion

CACP is a syndrome of precocious arthropathy that is initiated by a lack of the lubricating glycoprotein PRG4, secreted by synovial fibroblasts and superficial zone chondrocytes. PRG4 provides boundary lubrication on the articular cartilage surface [23,24]. The synovial fluid in patients with CACP syndrome is rich in hyaluronic acid [7], which by itself, similar to other synovial fluid constituents, is unable to provide full chondroprotection. Patients with CACP either do not express PRG4 or express a variant that does not function due to mutations in the mucin or hemopexin domain [2,25]. This preclinical study of rhPRG4 in *Prg4*^−/−^ mice offers our first insights into whether rhPRG4 shows activity in this inherited arthrosis in an orthologous animal model.

Describing the gait characteristics of *Prg4*^−/−^ mice is an important step to evaluate the functional implications of CACP and future therapeutic attempts. This investigation revealed that the phenotypic arthrosis resulting from the *Prg4*^−/−^ state from birth differs between male and female mice (Table 1). Some gait parameters only showed phenotypic differences in one sex but not the other, such as the decrease in stance time for *Prg4*^−/−^ females compared to *Prg4*^+/+^ female littermates, whereas other gait parameters were more similar between the sexes. The IA rhPRG4 increased the stance and propulsion time in *Prg4*^−/−^ male mice by day 3 following hindlimb knee injection (Table 2a). Female mice did not respond in a similar manner to the IA rhPRG4 (Table 2b). Six days after the IA rhPRG4, neither male nor female *Prg4*^−/−^ mice showed any effects on quadruped gait. These observations coincided with the reversal of caspase-3 activation in superficial zone chondrocytes in both sexes 10 days following IA injection (Figure 1). This observation also recapitulates a previous study of mitochondrial dysregulation in *Prg4*^−/−^ gene trap mice that also observed activation of caspase-3 in chondrocytes that was reversible upon conditional *Prg4* expression [8].

PRG4 works by lubricating articular surfaces, alleviating the mechanical shear of underlying superficial zone chondrocytes and thus lessening mitochondrial stresses, and ultimately caspase-3 activation. Caspase inhibitors have already been identified as having disease-modifying roles in human OA [26]. The present results are partly biophysical in nature through the alleviation of friction-induced exaggerated mechanical deformation upon mitochondria within superficial zone chondrocytes, which our laboratory [8,9] and others [27] have described. Deformation is exaggerated in high-friction states caused by stick-slip phenomenon at near zero low sliding speeds, forming adhesion bridges in the absence of Prg4, which may damage collagen at the surface of cartilage. The observed affinity of collagen hybridizing peptide to the superficial zone (Supplementary Figure S1) appears to support a mechanical damage mechanism [21], although remodeling [28] is also possible. Thus, the quartz microbalance was used to measure the surface activity of rhPRG4 as a change in the resonant frequency of a quartz crystal substrate. Albumin showed no effect, which is known to biofoul articular cartilage [6] in the *Prg4*^−/−^ joint in lieu of chondroprotective Prg4 (Figure 5). The rhPRG4 has a higher binding affinity than BSA [29] due to its surface-active nature, and likely explains why cartilage from *Prg4*^−/−^ mouse joints are characteristically fouled with a layer(s) of albumin and gamma globulin [3,6]. The observed in vitro biophysical activity at the hydrophilic–hydrophobic interface may recapitulate the SF–cartilage interface since the latter has a hydrophobic character [30]. The quartz microbalance experiments show that rhPRG4 binds avidly to an interface and does so despite pre-existing biofouling albumin, possibly recapitulating a similar effect on CACP cartilage surfaces. Monomerizing the rhPRG4 through disulfide bond reduction via DTT showed an even larger effect.

The pooled-sex articular cartilage RNA-Seq data indicates that the expression of Mt-tn was increased following IA rhPRG4 and is a component of the mitochondrial enzyme Complex 1 necessary for protein synthesis [31]. Surprisingly, myosin-related proteins, such as Myh7, and troponin subunits Tnnc1 and Tnnt1 were significantly upregulated (Figure 2 and Figure 3) following IA rhPRG4. This is important since non-muscle myosin heavy chains contribute to mitochondrial DNA maintenance [32]. Both Tnnc1 and Tnnt1 are also involved in organizing cytoskeletal actin, playing roles in cellular traction and migration [33]. Together, these cellular subunits, better known for their function in the sarcomere, may play a fundamental role in stabilizing the mitochondria. In addition, Troponin I1 (Tnni1) slow skeletal type has a known association with CACP in humans [34]. The GeneMANIA [35] co-expression and association analysis (Figure 3B and Figure S2) supports this conclusion since other subcellular and cytoskeletal genes are associated with mitochondria DNA, such as xin actin-binding repeat containing 1 (*Xirp1*) [36], mitochondrial biogenesis-like sarcolipin (*Sln*) [37], and ankyrin repeat domain 2 (*Ankrd2*) [38]. Other associated genes from the GeneMANIA analysis are caspase-3, which is central to our overarching hypothesis; mitochondrial creatinine kinase (*Ckmt2*) [39]; leucine-rich repeat; sterile alpha motif-containing 1 (*Lrsam 1*) [40]; and pleckstrin homology domain-containing family F (*Plekhf1*) [41], which are respectively involved in mitochondrial metabolism and cell death via the Plekhf1-dependent lysosomal-mitochondria pathway. The gene *Lars2*, enhanced by rhPRG4 in the female RNA-Seq data, is responsible for mitochondrial leucyl tRNA synthetase 2 expression [42].

The behavioral results of the present study parallel an earlier study of IA injection of hyaluronan for symptomatic relief in a murine model of OA [43] conducted in C57BL/6 male mice, aged 12 weeks, that also showed an acute and prolonged increase in swing and stance times by hyaluronan after the induction of OA. However, that study used a walking speed of 18 cm/s at a 17 degree uphill gradient, as opposed to the present study’s 28 cm/s at no incline. A model of rheumatoid arthritis in DBA/1LacJ mice showed significantly decreased swing, stride, stance, and propulsion time across more severe gradations of arthritis in female mice [44]. Among other studies that utilized DigiGait in a mutant mouse model, a shortened stance time was observed in mice lacking CX3CR1 resulting in phenotypic hip dysplasia [45]. A decrease in stance time was also observed in mice that received IA carrageenan, thus correlating decreased stance time with mechanical allodynia [46].

One of the main complaints from CACP patients is pain, with increased pain levels after age 10 [4]. Most patients are treated with NSAIDs and others may be treated with immunosuppressants or anti-inflammatory biologics, such as etanercept [25]. These treatments show little effect apart from mild pain relief [14]. In the present study, the IA injection of rhPRG4 decreased NGF expression by synoviocytes at day 10. The improved gait characteristic in male mice resulting from IA rhPRG4 injection may be associated with less nociceptive pain as reported in rodent studies [47,48]. The disparity in locomotor behavioral effects between male and female mice in experimental arthritis models has been observed before [49,50]. Nociceptive pain is sex dependent and related to the cross talk between afferent neurons in the synovium and infiltrating immune cells, such as macrophages [51]. Female rodents generate a greater immune response than male counterparts, involving both innate [52] and adaptive immunity [53]. Thus, females may experience more mechanical allodynia, generate less joint loading, and thus less cartilage degeneration occurs. This may also explain why at baseline, female *Prg4*^−/−^ mice (Table 1) demonstrated a shorter stance time than female *Prg4*^+/+^ and male *Prg4*^−/−^ littermates. In male rodents, innate immunity is TLR receptor dependent, where TLR antagonists suppressed mechanical allodynia in only male mice in nerve injury studies [53]. In the arthritic joint, aggrecan fragments are thought to produce pain through a TLR2 interaction [54]. This is particularly relevant for the present study since PRG4 is a TLR2 and TLR4 antagonist [55,56], and regulates synovial macrophage polarization by diminishing the numbers of synovial M1 macrophages [57].

The RNA recovered from the present lubricin-null joints shows that synovium from *Prg4*^−/−^ mice treated with rhPRG4 show more Bcl2 and less Cad (Figure 4), indicating stabilization of the mitochondria. The trending decrease of Bid by day 20 post-injection also suggests an anti-apoptotic action for the cell cycle, indicating that anastasis, the reversal of apoptosis, may have occurred. Traditionally, programmed cell death caused by apoptosis has been considered an irreversible process, involving the release of mitochondrial cytochrome *c* into the cytosol and the activation of execution caspases, such as caspase-3. However, emerging evidence suggests that cells undergoing programmed cell death can recover [58].

Despite the regulation of some pro-apoptotic and anti-apoptotic factors to reverse apoptosis, other anti-apoptotic factors, such as Parp and the heat shock proteins Hsp27, Hsp70 [59], and Hsp90, were downregulated in the synovium. Upstream, at the level of the apoptosis inducers, reactive oxygen species (ROS)-, reactive nitrogen species (RNS)-, and tumor necrosis factor α (TNFα)-induced chondrocyte apoptosis plays a major role [60]. Evidence for TNFα-induced chondrocyte apoptosis has been found in the pro-apoptotic action of TNF-related apoptosis-inducing ligand receptors (e.g., DR4 and DR5) both in vitro and in vivo. This was accompanied by active caspase-3 immunoreactivity, cytochrome c release, and cleavage of PARP [61,62]. The TNFα-induced chondrocyte apoptosis pathway is also associated with ROS generation in OA chondrocytes, as the mitochondrial protein TNFα-receptor-associated protein 1 (TRAP1), a member of the HSP90 family, was found in high abundance in those chondrocytes. The function of TRAP1 to antagonize ROS in protection against oxidative stress-induced apoptosis may signify the redox imbalance of the cellular environment [63]. Part of PRG4’s anti-apoptotic effects can also be attributed to inhibition of the TNFα-induced chondrocyte apoptosis pathway, as PRG4 is known to bind to CD44 to prevent the expression of TNFα [64].

ROS and RNS lead to mitochondrial dysfunction and are generated by stressed mitochondria. Peroxynitrite is a reaction product of nitric oxide and superoxide anions leading to induction of chondrocyte apoptosis [65,66]. Furthermore, ROS itself activates caspase-3-like proteases and caspase-3 [67]. When *Prg4* gene trap mice, postnatally in a *Prg4*^−/−^ state, were recombined to re-express Prg4 later in life, cartilage in their tibiofemoral joints had a significantly reduced peroxynitrite content and caspase-3-positive staining, compared to *Prg4*^−/−^ littermates [68]. ROS are associated with mitochondrial dysfunction as the electron transport chain slows with decreased activity at Complex I, II, and III, which may be implicated in excess chondrocyte generation of ROS and RNS [69,70]. As such, the chondroprotective effects of PRG4 may also be due to decreased mitochondrial DNA damage and increased repair [69]. PRG4, autophagy, and antioxidant defense could be linked, as one study found that FoxO transcription factors modulate these factors simultaneously and synergistically with transforming growth factor-β stimulation [71].

Limitations of this study revolve around studying a temporizing treatment in an animal model that already shows advanced disease. In addition, the vagaries of gender in arthritis animal models may have also occurred in this study. Potentially limiting the strength of the effect of IA rhPRG4 is the analysis of quadruped gait following bilateral hindlimb injections. However, the maturation of gait in mice studied in the DigiGait parallels normal human gait development in terms of the percent of stride in the stance and swing phase [72] and yet other DigiGait studies of monoarthritis have failed to show measurable changes in gait [73] but used a slower treadmill speed than the present study. We also do not know how much of the rhPRG4 was retained on the articular surfaces given the prolonged tissue phase half-life of rhPRG4 [74]. An immunohistochemical approach is not readily available since many of the immunoprobes were developed in mice [75] and cross reacted with the biofouling protein (data not shown).

In conclusion, the IA injection of rhPRG4 may offer some benefit to young patients with CACP judging by the correlation between inhibition of caspase-3 activation in chondrocytes and the observed quadruped gait improvement in stance and propulsion in male mice. The re-introduction of the missing functional protein in the orthologous murine disease model provides proof of principle of a therapeutic potential that is partially dependent on reversing mitochondrial dysfunction [76]. Patients with CACP typically seek rheumatologic evaluation given their multiple joint complaints. Currently, no treatments exist to restore this essential mucinous glycoprotein, although both helper-dependent virus [77] and adeno-associated virus [78] approaches exist and rhPRG4 has been used in a prior clinical trial [79]. There remains a need for more clinical information on the progression of CACP syndrome with advancing age both in childhood and beyond. In addition, information is lacking regarding the long-term consequences of heterozygosity for PRG4 mutations in the family members of affected individuals.

## 4. Materials and Methods

### 4.1. Prg4^−/−^ Mice

Mice with a mutant *Prg4* allele were created by homologous recombination in 129Sv/Ev-derived embryonic stem cells as described previously [6], resulting in the replacement of *Prg4* exon 6 with *lacZ* and *neo*. The genetic identity of progeny from the mating of *Prg4*^+/−^ and *Prg4*^+/−^ littermates was analyzed by a PCR-based genotype assay used previously [6]. Mice were euthanized following CO_2_ inhalation and their joints were removed and processed immediately after. All procedures for mouse research were approved by the Institutional Animal Care and Use Committee of Rhode Island Hospital. Here, 7- to 8-week-old *Prg4*^−/−^ mice (24 male and 20 female) and 7–8-week-old *Prg4*^+/+^ mice (9 male and 5 female) were used in gait, knee joint injection, and tissue studies. A random selection of these animals were euthanized 10 and 20 days following joint injection for histological studies and synoviocyte RNA collection. An additional 6 male and 6 female 7–8-week-old *Prg4*^−/−^ mice were used in the RNA-Seq gene expression studies.

### 4.2. Manufacture and Purification of rhPRG4

The expression of rhPRG4 by CHO-M cells has been described previously [80]. CHO-M cells were transfected with plasmid vectors (Selexis SA, Geneva, Switzerland) containing the human PRG4 gene, 1404 amino acids in length, under the control of hEF-1-alpha promoter coupled to a CMV enhancer [81]. The presence of O-linked glycosylations comprising (β1,3) Gal-GalNAc was previously confirmed by mass spectrometry [81]. rhPRG4 that was disulphide bond reduced (rhPRG4_DTT_) was prepared by incubating 2 mg/mL rhPRG4 with 10 mg/mL dithiothreitol for 2 h at 60 °C. rhPRG4 used in animal studies was filtered through a 0.22 μm filter prior to use.

### 4.3. In Vivo Tribosupplementation of Prg4^−/−^ Mouse Knees

For the 24 male and 20 female *Prg4*^−/−^ mice injected with either rhPRG4 or sterile PBS, the mice were anesthetized under isoflurane (3–5%), and a 70% alcohol wash used to prepare the site of injection. The knee was held in flexion and 10 μL of either rhPRG4 (2.0 mg/mL) or sterile PBS were injected via a 31G needle through the patellar tendon, confirmed by distension of the joint space. The needle was removed, and the knee was flexed and extended 10 times, distributing the injected fluid evenly throughout the joint cavity. Both hindlimbs were injected.

### 4.4. Gait Analysis

In this study, 7- to 8-week-old *Prg4*^−/−^ mice (24 male and 20 female) and 7–8-week-old *Prg4*^+/+^ mice (9 male and 5 female) were studied in the DigiGait gait analysis system (Mouse Specifics Inc, Framingham, MA, USA). The instrument allows mice to walk on a transparent treadmill belt. Underneath the belt a video camera captures the ventral side of the animal as it walks. DigiGait automatically detects and vectorizes the animal’s paws based on their pink coloration. The software uses image recognition and artificial intelligence algorithms to create a set of periodic waveforms describing the placement of the four limbs through consecutive strides. The software then analyzes the gait dynamics for 31 different postural and kinematic metrics of gait as described previously [82].

For each measurement collection, mice were ambulated at 28 cm/s with 0 degrees tilt for 30 s. Gait parameters included swing time (non-weight bearing phase of gait), percent of stride in swing, brake time, percent of stride in breaking, propulsion time, percent of stride in propulsion, stance time (weight bearing phase of gait), stance time/swing time, and stride time. The *Prg4*^−/−^ and *Prg4*^+/+^ mice were measured at baseline. The following day, half of the *Prg4*^−/−^ mice were randomly selected and injected IA in both hindlimbs with rhPRG4, and the other half of the *Prg4*^−/−^ mice were injected IA with PBS. Gait measurements were performed on days 3 and 6 post-injection by an operator blinded to the treatment identity.

### 4.5. Tissue Processing for Immunohistochemistry of Caspase-3 and Collagen Hybridizing Peptide

On day 10 post-injection with either rhPRG4 or PBS, knee joints were dissected out and fixed in 10% formalin (Fisher PROTOCOL^TM^, Thermo Fisher Scientific, Waltham, MA, USA) and decalcified using a solution of 0.48M EDTA, with an adjusted pH of 7.1 with ammonium hydroxide at 4 °C for 48 h. Coronal sections (5 μm), framing the femoral condyles and tibial plateau, were taken for histological analysis of caspase-3 activation. Sections were incubated at 60 °C for 30 min, deparaffinized, and hydrated in 3 cycles of xylene and serial alcohol. Incubation in a pepsin solution (Thermo Fisher Scientific, Waltham, MA, USA) allowed for antigen retrieval. A 1:1000 dilution of rabbit polyclonal antibody against active caspase-3 antibody 961 (Cell Signaling Technology, Danvers, MA, USA) was added for incubation at 4 °C overnight. After 3 washes with PBS, the sections were incubated for 1 h in 1:1000 dilution of Cy3 goat anti-rabbit IgG at room temperature (Life Technologies, Molecular Probes, Eugene, OR, USA). After another five washes with PBS, the sections were counterstained using Vectashield mounting media with DAPI (Vector Laboratories Inc., Burlingame, CA, USA). Adjacent sections were also probed with collagen hybridizing peptide (3Helix) [21,83] using the manufacturer’s recommendations, imaged using Image Pro 9.3 (Media Cybernetics, Rockville, MD, USA), and counted as described previously [84].

### 4.6. Quantitative Real-Time Polymerase Chain Reaction of Synovial RNA

First, 2 groups of N = 3 mice from the above rhPRG4- and PBS-injected animals were euthanized separately 10 and 20 days following IA injection. Messenger RNA (mRNA) was isolated from the knee synovium of both hindlimbs following homogenization in 2 60 s periods at 60 Hz using a KZII homogenizer (Servicebio, Wuhan, China) interspersed by a 60 s incubation at 4 °C. RNA was extracted and purified using an RNeasy mini kit (Qiagen, Germantown, MD, USA). Total RNA was obtained for each group of 3 mice and measured by a Nanodrop 2000c (Thermo Scientific, Waltham, MA, USA). The samples were pooled by injection group for qRT-PCR analysis using the cDNA primers summarized in Supplementary Table S3 and manufactured by MilliporeSigma (Burlington, MA, USA). The cycle threshold (Ct) value of genes of interest was normalized to the Ct value for GAPDH in the same sample and the relative expression was calculated using the 2^−ΔΔCt^ method [85].

### 4.7. RNA-Seq Analysis of Chondrocyte RNA

The additional 6 male and 6 female *Prg4*^−/−^ mice described above were injected with rhPRG4 or PBS by block randomization, which resulted in 2 rhPRG4 and 2 PBS groups of 3 mice each sharing the same sex. Ten days later, cartilage was obtained and pooled from three mice sharing the same sex and IA treatment. The cartilage was homogenized in 2 60 s periods at 60 Hz using a KZII homogenizer (Servicebio, Cambridge, MA, USA) interspersed by a 60 s incubation at 4 °C. RNA was extracted and purified using an RNeasy mini kit (Qiagen, Germantown, MD, USA). Total RNA was obtained for each group of 3 mice and measured by a Nanodrop 2000c (Thermo Scientific, Waltham, MA, USA). In total, 5 μg of total RNA from each group were used for RNA-Seq analysis performed by GENEWIZ (South Plainfield, NJ, USA), who were blinded to the group identity. Library preparation completed at GENEWIZ utilized PolyA selection and rRNA depletion. Furthermore, 150 bp paired-end sequencing was performed on an Illumina HiSeq (Illumina Inc., San Diego, CA, USA).

Sequence reads were trimmed to remove possible adapter sequences and nucleotides with poor quality using Trimmomatic v.0.36 (Anthony Bolga and Bjorn Usadel, Aachen, Germany). The trimmed reads were mapped to the *Mus musculus* GRCm38 reference genome available on ENSEMBL using the STAR aligner v.2.5.2b (Alex Doblin, Cold Spring Harbor Laboratory, Cold Spring Harbor, NY, USA) and BAM files were generated. Unique gene hit counts were calculated by using feature counts from the Subread package v.1.5.2 (Alex Doblin, Cold Spring Harbor Laboratory, Cold Spring Harbor, NY, USA). The hit counts were summarized and reported using gene_id and only unique reads that fell within exon regions were counted. Since a strand-specific library preparation was performed, the reads were strand-specifically counted.

After the extraction of gene hit counts, a gene hit counts table was used for downstream differential expression analysis. Using DESeq2, a comparison of the gene expression between rhPRG4 and PBS in both sexes was performed. Confirmatory qRT-PCR was performed and relative fold changes for both IA rhPRG4- and PBS-injected limbs were calculated using the 2^−ΔΔCt^ method [85] on identified upregulated genes of interest from the analysis of both sexes. Supplementary Table S3 illustrates the cDNA primers used. The primer pairs for *Xist* (Cell Signaling Technology) and Lars2 (OriGene, Rockville, MD, USA) were sourced commercially. The primer sequence for *Myh7* was used previously [86].

### 4.8. Gene Ontology Analysis

A gene ontology analysis was performed on the statistically significant set of genes by implementing the software GeneSCF v.1.1-p2 (Santhilal Subhash, Cold Spring Harbor Laboratory, Cold Spring Harbor, NY, USA). The mgi GO list was used to cluster a set of genes based on their biological processes and determine their statistical significance. A list of clustered genes was generated based on their gene ontologies.

### 4.9. Adsorption and Anti-Adhesion by rhPRG4 Using Quartz Crystal Microbalance

The non-specific adsorption and anti-adhesive properties of rhPRG4 were measured using QCM 200 (Stanford Research Systems, Sunnyvale, CA, USA) as described by Greene et al. [87]. This method consisted of the following steps: a quartz crystal microbalance Cr/Au 5 MHz (part#6-613, Stanford Research Systems) wafer was equilibrated with PBS at a constant flow rate of 300 μL/min; either 1 mL of rhPRG4 (diluted with PBS to a concentration of 300 μg/mL) or bovine serum albumin (BSA) (300 μg/mL) was infused into the QCM flow cell; a 20 min incubation followed, after which the crystal was equilibrated with PBS until a stable frequency baseline was achieved; either 1 mL of 300 μg/mL of BSA or rhPRG4 was introduced followed by another 20 min incubation and PBS equilibration. The difference in frequency, ΔF (measure of adsorption), of the QCM crystal was measured using a customized MATLAB algorithm. ΔF values for BSA on a rhPRG4- or rhPRG4_DTT_-coated surface and vice versa were determined. Moreover, ΔF values for BSA, rhPRG4, and rhPRG4_DTT_ were obtained using control experiments, wherein the steps were the same except that the samples were followed by 1 mL of PBS.

### 4.10. Statistics

Hindlimb gait measurements were compared between *Prg4*^−/−^ and *Prg4*^+/+^ mice using two-way analyses of variance with type (*Prg4*^−/−^ vs. *Prg4*^+/+^) and sex as fixed factors. Comparisons between *Prg4*^−/−^ mice injected with rhPRG4 and those injected with PBS were based on mixed model repeated measures analyses with group and time as fixed factors and animal as a random factor. Post-hoc tests corresponding to pre-planned comparisons were based on linear contrasts comparing changes from baseline to post-injection days 3 and 6 between groups for both sexes. Statistical significance was determined based on *p* < 0.05. Analyses were performed using SAS Statistical Software Version 9.4 (SAS Institute, Cary, NC, USA).

The Wald test was used to generate *p*-values and log2 fold changes for expressed genes quantified by RNA-Seq. Significantly differentially expressed genes quantified by RNA-Seq were identified if the *p*-value was <0.05 and upregulation was greater than 2.3 or downregulation greater than 0.5 on a log2 fold change scale. Three analysis groups were pooled for gene expression identification: Female PBS vs. Female rhPRG4; Male PBS vs. Male rhPRG4; and Both sexes PBS vs. rhPRG4. R studio (Boston, MA, USA) was used to create heatmaps using the “pheatmap” package. GraphPad Prism (San Diego, CA, USA) was used to illustrate log2 fold changes in differential genes, log10 fold changes in fluorescence intensity, and statistical differences in the numbers of activated caspase-3-positive chondrocytes between PBS- and rhPRG4-injected joints. A Volcano plot of log10 of the adjusted *p*-value against the log2 fold change in differentially expressed genes was constructed in the R open source statistical computing software.

## Figures and Tables

**Table 1:** Difference in hindlimb gait between *Prg4*^−/−^ and *Prg4*^+/+^ mice by sex.

	Male Mean (SD)		Female Mean (SD)	
**PSwingStride (%)**				
Percent of the stride in the swing phase				
*Prg4* ^−/−^	37.1	(2.1)	38.0	(1.3)
*Prg4* ^+/+^	36.1	(1.3)	39.7	(1.4)
*p*-value (Group differences within Sex)	0.198		0.098	
*p*-value (Group by Sex Interaction)	0.038			
**PBrakeStride (%)**				
Percent of the stride in the brake phase				
*Prg4* ^−/−^	12.5	(2.5)	13.4	(1.8)
*Prg4* ^+/+^	19.5	(3.9)	15.4	(1.6)
*p*-value (Group differences within Sex)	<0.001		0.107	
*p*-value (Group by Sex Interaction)	0.003			
**PPropelStride (%)**				
Percent of the stride in the propel phase				
*Prg4* ^−/−^	50.4	(2.5)	48.6	(2.3)
*Prg4* ^+/+^	44.4	(4.1)	44.9	(1.1)
*p*-value (Group differences within Sex)	<0.001		0.007	
*p*-value (Group by Sex Interaction)	0.184			
**Stance/Swing**				
Stance time divided by swing time				
*Prg4* ^−/−^	1.71	(0.15)	1.65	(0.16)
*Prg4* ^+/+^	1.78	(0.10)	1.51	(0.08)
*p*-value (Group differences within Sex)	0.247		0.059	
*p*-value (Group by Sex Interaction)	0.029			
**Swing Time (s)**				
The time for forward portion of the stride in which the paw is not in contact with the belt				
*Prg4* ^−/−^	0.077	(0.015)	0.089	(0.004)
*Prg4* ^+/+^	0.072	(0.006)	0.072	(0.006)
*p*-value (Group differences within Sex)	0.048		<0.001	
*p*-value (Group by Sex Interaction)	<0.001			
**Stance Time (s)**				
The time for portion of stride where the paw remains in contact with the belt				
*Prg4* ^−/−^	0.131	(0.008)	0.118	(0.008)
*Prg4* ^+/+^	0.127	(0.007)	0.134	(0.005)
*p*-value (Group differences within Sex)	0.185		<0.001	
*p*-value (Group by Sex Interaction)	<0.001			
**Stride Time (s)**				
The amount of time needed to complete one full stride for one limb				
*Prg4* ^−/−^	0.208	(0.016)	0.190	(0.011)
*Prg4* ^+/+^	0.198	(0.012)	0.223	(0.006)
*p*-value (Group differences within Sex)	0.058		<0.001	
*p*-value (Group by Sex Interaction)	<0.001			
**Brake Time (s)**				
The time between initial paw contact with the belt and the maximal paw contact				
*Prg4* ^−/−^	0.026	(0.004)	0.025	(0.003)
*Prg4* ^+/+^	0.039	(0.008)	0.034	(0.004)
*p*-value (Group differences within Sex)	<0.001		<0.001	
*p*-value (Group by Sex Interaction)	0.186			
**Propel Time (s)**				
Time between maximal paw contact and the end of the stance, just before swing				
*Prg4* ^−/−^	0.105	(0.010)	0.093	(0.009)
*Prg4* ^+/+^	0.088	(0.009)	0.100	(0.003)
*p*-value (Group differences within Sex)	<0.001		0.106	
*p*-value (Group by Sex Interaction)	<0.001			

Male (*Prg4*^−/−^ N = 24, *Prg4*^+/+^ N = 9); Female (*Prg4*^−/−^ N = 20, *Prg4*^+/+^ N = 5). *p*-values correspond to test for group difference in change from baseline. rhPRG4 Injected N = 22; PBS Injected N = 21.

**Table 2:** (a). Male hindlimb gait data from baseline to 6 days post-injection. (b). Female hindlimb gait data from baseline to 6 days post-injection.

**(a)**						
	**Males**					
	**Baseline** **Mean (SD)**		**Day 3** **Mean (SD)**		**Day 6** **Mean (SD)**	
**PSwingStride (%)**						
rhPRG4 Injected	37.8	2.2	36.6	2.6	36.6	2.2
PBS Injected	36.4	1.8	36.5	1.2	36.8	1.8
*p*-value			0.134		0.077	
**PBrakeStride (%)**						
rhPRG4 Injected	12.4	2.1	12.2	1.7	12.3	2.8
PBS Injected	12.6	2.9	13.1	2.4	12.0	2.2
*p*-value			0.544		0.726	
**PPropelStride (%)**						
rhPRG4 Injected	49.8	2.2	51.3	3.2	51.1	2.4
PBS Injected	51.0	2.7	50.4	1.5	51.2	2.9
*p*-value			0.152		0.441	
**Stance/Swing**						
rhPRG4 Injected	1.66	0.15	1.76	0.23	1.74	0.16
PBS Injected	1.76	0.14	1.75	0.10	1.73	0.13
*p*-value			0.099		0.099	
**Swing Time (s)**						
rhPRG4 Injected	0.079	0.009	0.078	0.009	0.074	0.007
PBS Injected	0.076	0.010	0.073	0.005	0.074	0.010
*p*-value			0.742		0.204	
**Stance Time (s)**						
rhPRG4 Injected	0.130	0.006	0.134	0.007	0.127	0.004
PBS Injected	0.131	0.010	0.127	0.006	0.127	0.007
*p*-value			0.005		0.756	
**Stride Time (s)**						
rhPRG4 Injected	0.210	0.013	0.212	0.013	0.200	0.010
PBS Injected	0.206	0.019	0.200	0.010	0.200	0.017
*p*-value			0.080		0.517	
**Brake Time (s)**						
rhPRG4 Injected	0.026	0.004	0.026	0.004	0.025	0.005
PBS Injected	0.026	0.005	0.026	0.004	0.024	0.005
*p*-value			0.835		0.876	
**Propel Time (s)**						
rhPRG4 Injected	0.104	0.008	0.109	0.008	0.102	0.006
PBS Injected	0.105	0.012	0.101	0.006	0.103	0.007
*p*-value			0.020		0.848	
**(b)**						
	**Females**					
	**Baseline** **Mean (SD)**		**Day 3** **Mean (SD)**		**Day 6** **Mean (SD)**	
**PSwingStride (%)**						
rhPRG4 Injected	38.3	2.6	37.5	1.3	37.9	2.0
PBS Injected	37.8	2.1	38.5	1.3	38.2	2.4
*p*-value			0.154		0.401	
**PBrakeStride (%)**						
rhPRG4 Injected	13.0	1.8	13.8	3.1	14.6	2.4
PBS Injected	13.5	1.8	12.5	3.1	13.1	1.9
*p*-value			0.240		0.203	
**PPropelStride (%)**						
rhPRG4 Injected	48.7	2.5	48.7	2.4	47.5	2.2
PBS Injected	48.7	2.2	49.0	3.1	48.6	2.6
*p*-value			0.860		0.412	
**Stance/Swing**						
rhPRG4 Injected	1.63	0.18	1.67	0.09	1.65	0.15
PBS Injected	1.66	0.15	1.60	0.09	1.64	0.17
*p*-value			0.202		0.591	
**Swing Time (s)**						
rhPRG4 Injected	0.072	0.006	0.072	0.004	0.071	0.009
PBS Injected	0.073	0.006	0.076	0.007	0.074	0.008
*p*-value			0.388		0.476	
**Stance Time (s)**						
rhPRG4 Injected	0.116	0.008	0.120	0.004	0.116	0.011
PBS Injected	0.120	0.009	0.121	0.007	0.119	0.009
*p*-value			0.522		0.884	
**Stride Time (s)**						
rhPRG4 Injected	0.189	0.010	0.192	0.007	0.187	0.019
PBS Injected	0.193	0.012	0.196	0.013	0.193	0.013
*p*-value			0.968		0.770	
**Brake Time (s)**						
rhPRG4 Injected	0.025	0.003	0.026	0.005	0.027	0.004
PBS Injected	0.026	0.002	0.024	0.005	0.025	0.003
*p*-value			0.189		0.207	
**Propel Time (s)**						
rhPRG4 Injected	0.092	0.008	0.093	0.007	0.089	0.010
PBS Injected	0.094	0.009	0.096	0.010	0.094	0.009
*p*-value			0.904		0.544	

*p*-values correspond to test for male and female group difference in change from baseline. rhPRG4 injected N = 12 male and N = 10 female; PBS injected N = 12 male and N = 9 female.

## Data Availability

Data is publicly available on Zenodo site which can be accessed through https://doi.org/10.5281/zenodo.6426032 (accessed on 30 March 2022).

## Supplementary Materials

The following supporting information can be downloaded at: https://www.mdpi.com/article/10.3390/ijms23084245/s1.
