# Supplementary material for: Quadruped Gait and Regulation of Apoptotic Factors in Tibiofemoral Joints following Intra-Articular rhPRG4 Injection in Prg4 Null Mice
Source: Int J Mol Sci. 2022 Apr 12;23(8):4245. doi: 10.3390/ijms23084245 (PMC9025840; doi:10.3390/ijms23084245)
Supplement: Supplementary file 1 [file ijms-23-04245-s001.zip › ijms-1685596-supplementary.pdf]

Supplemental Table S1. Mapped RNA-Seq reads of *M. musculus* genome

Sample ID	Total Reads	Total Mapped Reads	% Total Mapped Reads	Unique Mapped Reads	% Unique Mapped Reads
LUBF1	36590775	25118712	68.64	15881169	43.40
LUBF2	33448004	19834982	59.30	15180440	45.38
LUBF3	27161382	17669437	65.05	13605463	50.09
LUBM1	34400855	22371720	65.03	16154568	46.96
LUBM2	36315874	23848286	65.67	18141147	49.95
LUBM3	29299012	23200913	79.18	18016334	61.49
PBSF1	39116779	22193698	56.73	14087055	36.01
PBSF2	44562009	21537723	48.33	13867401	31.12
PBSF3	44795502	22244019	49.65	14647927	32.69
PBSM1	32616338	20779813	63.71	15984367	49.00
PBSM2	30432361	21523578	70.72	16808787	55.23
PBSM3	33377599	24679298	73.94	19036823	57.03

Supplemental Table S2. Differentially Regulated Genes Identified by RNA-Seq. Green (Upregulated) & Red (Downregulated)

Symbol	Gene name	Gene Function
	**Upregulated & Downregulated Genes in Male Mice rhPRG4 vs PBS**	
Scube 1	Signal peptide, CUB domain and EGF like domain containing 1	Protein Expression
Wif1	WNT Inhibitory Factor 1	Cell Signaling
Matn3	Matrilin 3	Extracellular matrix fibrillar protein
Cxcl14	C-X-C Motif Chemokine Ligand 14	Secreted cytokine
Hapln1	Hyaluronan And Proteoglycan Link Protein 1	Extracellular matrix
Mss51	MSS51 Mitochondrial Translational Activator	Mitochondrial protein expression
Col2a1	Collagen Type II Alpha 1 Chain	Extracellular matrix fibrillar protein
Tnn	Tenascin N	Extracellular matrix glycoprotein
Prg2	Proteoglycan 2, Pro Eosinophil Major Basic Protein	Extracellular carbohydrate binding
Sfrp2	Secreted Frizzled Related Protein 2	Modulator of Wnt Signaling
Coll2a1	Collagen 2A1	Extracellular matrix fibrillar protein
Fam129c	Family with sequence similarity 129 member C	Regulates p53-mediated apoptosis
Mmp13	Matrix Metallopeptidase 13	Protease of extracellular matrix
Myh7	Myosin Heavy Chain 7	Extracellular matrix and ATPase
Kdm5d	Lysine Demethylase 5D	Histone H3 Protein Processing
Uty	Ubiquitously Transcribed Tetratricopeptide Repeat Containing, Y-Linked	Histone H3 Protein Processing
Ddx3y	DEAD-Box Helicase 3 Y-Linked	ATP-dependent RNA helicase
Eif2s3y	Eukaryotic translation initiation factor 2 subunit 3, Y-linked	Heterotrimeric GTP-binding protein
Amd1	Adenosylmethionine Decarboxylase 1	Enzyme in polyamine biosynthesis
Igkc	Immunoglobulin Kappa Constant	Secreted glycoprotein
Gbp4	Guanylate Binding Protein 4	Hydrolysis of GTP to GDP and GMP
Xist	X Inactive Specific Transcript	RNA gene involved in X inactivation
	**Upregulated & Downregulated Genes in Female Mice rhPRG4 vs PBS**	
My12	Myosin Light Chain 2	Extracellular matrix and ATPase
Matn3	Matrilin 3	Extracellular matrix fibrillar protein
Rgs9	Regulator Of G Protein Signaling 9	GTPase activating protein
Tnni1	Troponin I1, Slow Skeletal Type	Slow skeletal Actin binding protein
Hmgn5	High Mobility Group Nucleosome Binding Domain 5	Nuclear protein in transcription
Alox12b	Arachidonate 12-Lipoxygenase, 12R Type	Enzyme for Arachidonic acid
Lars2	Leucyl-tRNA Synthetase 2, Mitochondrial	Mitochondrial tRNA synthetase
Ccdc33	Coiled-Coil Domain Containing 33	Protein expression
Map7d2	MAP7 Domain Containing 2	Protein expression
Pkhd1	PKHD1 Ciliary IPT Domain Containing Fibrocystin/Polyductin	Transmembrane protein
Pou4f1	POU Class 4 Homeobox 1	Transcription factor
Igfn1	Immunoglobulin Like And Fibronectin Type III Domain Containing 1	Secreted protein expression
Cytl1	Cytokine Like 1	Protein expression
Mt-tv	Mitochondrially Encoded TRNA-Val (GUN)	tRNA affiliated gene
Mt-t12	Mitochondrially Encoded TRNA-Leu (CUN) 2	tRNA affiliated gene
Mt-tp	Mitochondrially Encoded TRNA-Pro (CCN)	tRNA affiliated gene
Snora73a	Small Nucleolar RNA, H/ACA Box 73A	snoRNA affiliated gene
Snora23	Small Nucleolar RNA, H/ACA Box 23	snoRNA affiliated gene
Snord118	Small Nucleolar RNA, C/D Box 118	snoRNA affiliated gene
Snora21	Small Nucleolar RNA, H/ACA Box 21	snoRNA affiliated gene
Rny3	RNA, Ro60-Associated Y3	Y_RNA affiliated gene
Snord104	Small Nucleolar RNA, C/D Box 104	noRNA affiliated gene
Vaultrc5	vault RNA component 5	Vault_RNA class affiliated gene
Rnu12	RNA, U12 Small Nuclear	snRNA affiliated gene
Rny1	RNA, Ro60-Associated Y1	Expressed ribonucleoprotein
Snord15a	Small Nucleolar RNA, C/D Box 15A	snoRNA affiliated gene
Snora57	Small Nucleolar RNA, H/ACA Box 57	snoRNA affiliated gene
Snora17	Small Nucleolar RNA, H/ACA Box 17	snoRNA affiliated gene
Snord17	Small Nucleolar RNA, C/D Box 17	snoRNA affiliated gene
Snord13	Small Nucleolar RNA, C/D Box 13	snoRNA affiliated gene
Snora78	Small Nucleolar RNA, H/ACA Box 78	snoRNA affiliated gene
Scarna6	Small Cajal Body-Specific RNA 6	snoRNA affiliated gene
Scarna3a	Small Cajal Body-Specific RNA	scaRNA affiliated gene
Asmt	Acetylserotonin O-Methyltransferase	Secreted enzyme
	**Upregulated Genes in Pooled Sex Mice PBS vs rhPRG4**	
Myh7	Myosin heavy chain 7	Extracellular matrix and ATPase
Mt-tn	Mitochondrially Encoded TRNA-Asn (AAU/C)	tRNA affiliated gene
Gm25747	Predicted Gene	
Igfn1	Immunoglobulin Like And Fibronectin Type III Domain Containing 1	Secreted protein expression
Myl2	Myosin light chain 2	Extracellular matrix and ATPase
Myl3	Myosin light chain 3	Extracellular matrix and ATPase
Tnnc1	Troponin C1, slow skeletal and cardiac type	Slow skeletal Actin binding protein
Tnni1	Troponin I1, slow skeletal type	Slow skeletal Actin binding protein
Tnnt1	Troponin T1, slow skeletal type	Actin binding & calcium metabolism

Supplemental Table S3. cDNA Primer Sequences Used in qRT-PCR

Gene	Description	Primer	Sequence (5' – 3')	Size (BP)
Xiap	X-linked inhibitor of apoptosis	F	CTGGCCGGACTATGCTCATT	
		R	CACGATCACAGGGTTCCCAA	131
Bid	BH3 interacting domain death agonist	F	CCTGTGCAAGCTTACTGGGA	
		R	TTTGTCTTCCTCCGACAGGC	243
Bcl2	B cell leukemia/lymphoma 2	F	CTTTGAGTTCGGTGGGGTCA	
		R	AGTTCCACAAAGGCATCCCA	153
Gapdh	Glyceraldehyde-3-phosphate dehydrogenase	F	GGAGAGTGTTTCCTCGTCCC	
		R	ACTGTGCCGTTGAATTTGCC	202
Cad	Carbamoyl-phosphate synthetase 2, aspartate transcarbamylase, and dihydroorotase	F	GAGCCCTACATAGCGCCTG	
		R	CAGCAGCTTTTGCCTCAGTG	306
Icad	Inhibitor of caspase-activated DNase	F	CAGGCCTTGGAGAAAGAGGG	
		R	TCTGGCTGTGTGAAAAGGCA	333
Hsp70	Heat shock protein 70 binding	F	CGACCTGAACAAGAGCATCA	
		R	ATGACCTCCTGGCACTTGTC	500*
Hsp27	Heat shock protein 27 binding	F	CCTCTTCGATCAAGCTTTCG	
		R	CTCAGGGGATAGGGAAGAGG	300*
Hsp90	Heat shock protein 90 binding	F	GAACATTGTGAAGAAGTGCC	
		R	GAACATTGTGAAGAAGTGCC	320
Parp1	Poly (ADP-ribose) polymerase family, member 1	F	GACACCCCGTGCAGACTTG	
		R	CCACCACACGTACTGCTCG	186
Ngf	Nerve growth factor	F	GCAGCATGGTGGAGTTTTGG	
		R	CTGTGTACGGTTCTGCCTGT	215
Myh7	Myosin heavy chain 7	F	CTCAAGCTGCTCAGCAATCTATTT	
		R	GGAGCGCAAGTTTGTCATAAGT	NA
Lars2	Leucyl-tRNA synthetase 2, mitochondrial	F	GCCTTCCATCTGGACAGTGTCT	
		R	GGACTTGCTCATCTTCTCCCAC	NA
Myl2	Myosin light chain 2	F	TCCCTGAGTGGGTTTGGGT	
		R	TTTGCCCTCGGGATCAAACA	416
Tnni1	Troponin I1	F	GCTCCACGAGGACTAAACTAGG	
		R	GAATGCGCTCCGAGAGGTAA	236

Size in base pairs (BP) is predicted unless indicated* and measured from agarose electrophoresis. Not available (NA) refers to a commercial or academic source described in Materials and Methods with an unknown amplicon size.

Supplemental Figure S1.

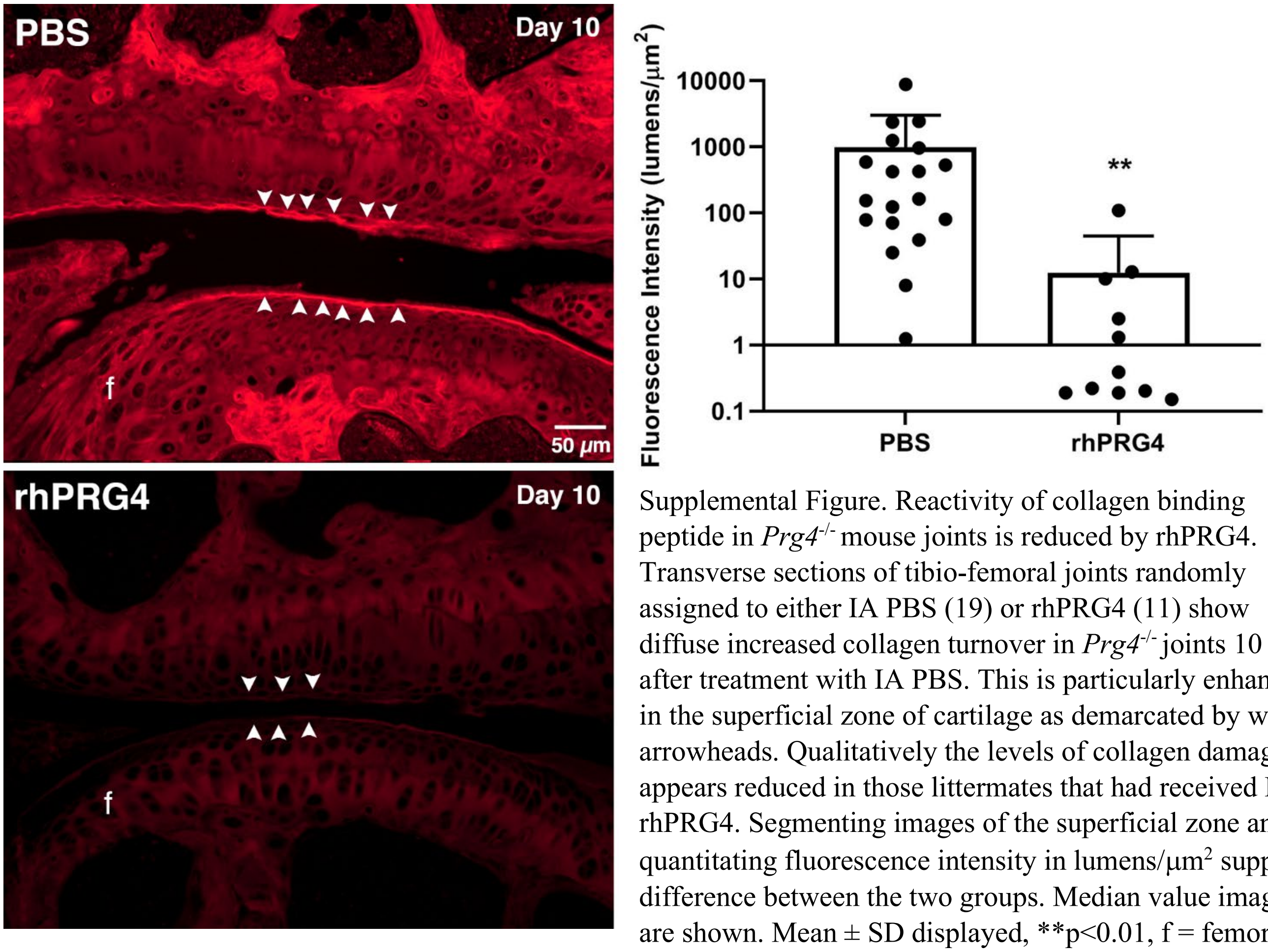

Supplemental Figure. Reactivity of collagen binding peptide in *Prg4*$^{-/-}$ mouse joints is reduced by rhPRG4. Transverse sections of tibio-femoral joints randomly assigned to either IA PBS (19) or rhPRG4 (11) show diffuse increased collagen turnover in *Prg4*$^{-/-}$ joints 10 days after treatment with IA PBS. This is particularly enhanced in the superficial zone of cartilage as demarcated by white arrowheads. Qualitatively the levels of collagen damage in appears reduced in those littermates that had received IA rhPRG4. Segmenting images of the superficial zone and quantitating fluorescence intensity in lumens/μm^2 support a difference between the two groups. Median value images are shown. Mean ± SD displayed, **$p<0.01$, f = femoral.

Supplemental Figure S2.

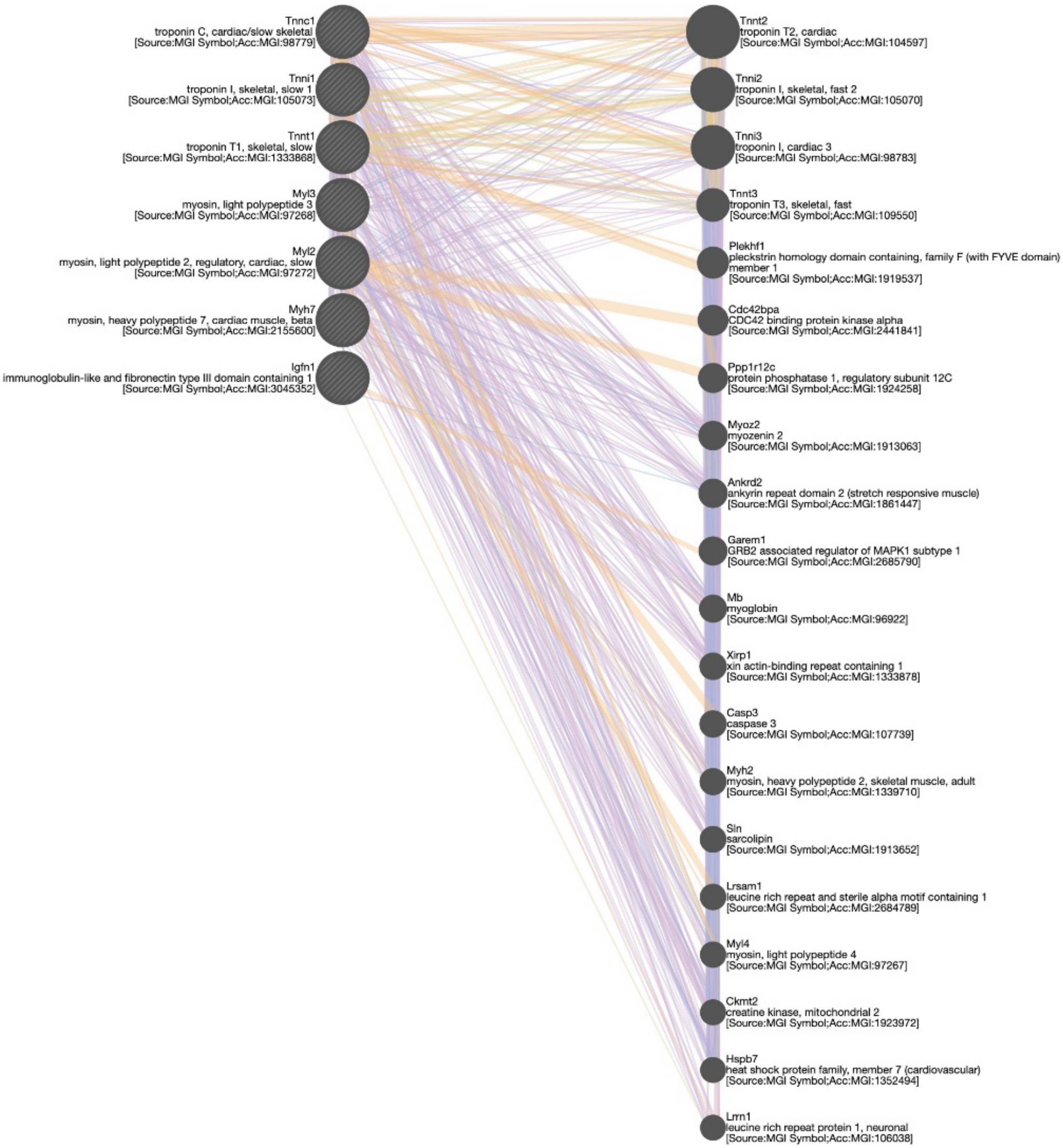

Supplemental Figure. GeneMANIA interaction network associated with Fig 3 where individual genes have been identified.
